# Can digitally enabling community health and nutrition workers improve services delivery to pregnant women and mothers of infants? Quasi-experimental evidence from a national-scale nutrition programme in India

**DOI:** 10.1136/bmjgh-2021-007298

**Published:** 2022-07-14

**Authors:** Sumeet R Patil, Sneha Nimmagadda, Lakshmi Gopalakrishnan, Rasmi Avula, Sumati Bajaj, Nadia Diamond-Smith, Anshuman Paul, Lia Fernald, Purnima Menon, Dilys Walker

**Affiliations:** 1 Center for Causal Research and Evaluations, NEERMAN, Mumbai, India; 2 Department of Economics, University of Southern California, Los Angeles, CA, USA; 3 Department of Health Policy, School of Public Health, UC Berkeley, Berkeley, CA, USA; 4 International Food Policy Research Institute, New Delhi, India; 5 Epidemiology and Biostatistics, University of California San Francisco, San Francisco, California, USA; 6 Community Health Sciences, School of Public Health, UC Berkeley, Berkeley, CA, USA; 7 Department of Obstetrics Gynecology and Reproductive Sciences, University of California San Francisco, San Francisco, California, USA; 8 Global Health Sciences, University of California San Francisco, San Francisco, California, USA

**Keywords:** health systems evaluation, nutrition, maternal health, child health, health services research

## Abstract

**Background:**

India’s 1.4 million community health and nutrition workers (CHNWs) serve 158 million beneficiaries under the Integrated Child Development Services (ICDS) programme. We assessed the impact of a data capture, decision support, and job-aid mobile app for the CHNWs on two primary outcomes—(1) timeliness of home visits and (2) appropriate counselling specific to the needs of pregnant women and mothers of children <12 months.

**Methods:**

We used a quasi-experimental pair-matched controlled trial using repeated cross-sectional surveys to evaluate the intervention in Bihar and Madhya Pradesh (MP) separately using an intention-to-treat analysis. The study was powered to detect difference of 5–9 percentage points (pp) with type I error of 0.05 and type II error of 0.20 with endline sample of 6635 mothers of children <12 months and 2398 pregnant women from a panel of 841 villages.

**Results:**

Among pregnant women and mothers of children <12 months, recall of counselling specific to the trimester of pregnancy or age of the child as per ICDS guidelines was higher in both MP (11.5pp (95% CI 7.0pp to 16.0pp)) and Bihar (8.0pp (95% CI 5.3pp to 10.7pp)). Significant differences were observed in the proportion of mothers of children <12 months receiving adequate number of home visits as per ICDS guidelines (MP 8.3pp (95% CI 4.1pp to 12.5pp), Bihar: 7.9pp (95% CI 4.1pp to 11.6pp)). Coverage of children receiving growth monitoring increased in Bihar (22pp (95% CI 0.18 to 0.25)), but not in MP. No effects were observed on infant and young child feeding practices.

**Conclusion:**

The at-scale app integrated with ICDS improved provision of services under the purview of CHNWs but not those that depended on systemic factors, and was relatively more effective when baseline levels of services were low. Overall, digitally enabling CHNWs can complement but not substitute efforts for strengthening health systems and addressing structural barriers.

**Trial registration number:**

ISRCTN83902145.

**What is already known on this topic?**

- Prior research from well-controlled pilot evaluations suggests that digital health interventions can enable community health and nutrition workers (CHNWs) deliver services better, but their impact on maternal and child nutrition practices is mixed.
- There is a dearth of evidence on effectiveness of digitally enabling community health workers of regional or national scale health and nutrition programmes in low-income and middle-income countries, and almost none when CHNWs are employed by the government.

**What this study adds**

- We find that digitally enabling CHNWs with a data capture, decision support and job-aid mobile app significantly improved the delivery of services within the sphere of control of CHNWs including home visits and counselling to mothers of children <12 months.
- Digitally enabling CHNWs is more effective when the baseline levels of services are low, but not when base-level of service is already high in regions with stronger public health systems (eg, growth monitoring of children in Madhya Pradesh vs Bihar states).
- The improvements in CHNW services did not translate into better infant and young child feeding practices unlike the results of past pilot-scale evaluations of similar digital interventions in India.

**How this study might affect research, practice and/or policy**

- Technology can enable CHNWs to improve delivery of services to a limited extent. However, digital health interventions cannot substitute the need for larger sectoral and programmatic reforms and interventions to address sociobehavioural barriers to achieving health and nutrition outcomes.
- National-scale public health programmes should do a careful assessment to understand whether, where, and how digital health interventions can help, and equally importantly, recognise what digital health interventions cannot deliver in silos.

## Introduction

Undernutrition is a major public health challenge worldwide, and has significant effects on birth weight, mortality, brain development and future earnings.^1–5^ Scaling up nutrition interventions to cover large segments of the population requires significant human, organisational and financial resources.^6^ In low-income and middle-income countries (LMICs), community health and nutrition workers (CHNWs) are considered essential for providing basic door-step services for nutrition, family planning and immunisation, and for monitoring and maintaining records of service delivery.^7^ Despite long-standing programmes that involve CHNWs, the evidence on the challenges faced by CHNWs to deliver these services effectively has accumulated around the world and in India.^8 9^

Digital health interventions—often grouped under a broad umbrella term ‘mHealth’—have the potential to support CHNWs to improve service coverage and quality^10^ and can even change health behaviours and improve health outcomes.^11^ However, evidence on how digital technology can help is limited to a handful of feasibility studies and efficacy trials of small-scale pilots.^12–17^ A recent systematic review noted that mobile phones were useful for promptness of data collection, surveillance and reduced errors by CHNWs.^12^ Another review found that technology supported better service delivery for antenatal visits, skilled birth attendance and postnatal visits.^16^ Another systematic review found that digital health interventions are correlated with increased interactions between clients (young mothers) and healthcare workers antenatally, during delivery, and postnatally in LMICs.^18^ However, the lack of robust evidence on large-scale effectiveness of digital health interventions in LMICs has been noted in all reviews. Only one recent study evaluated a nation-wide intervention (a Short Message Service [SMS] based data reporting to the health monitoring system) in Rwanda, but did not include a valid counterfactual analysis and was based on secondary public health monitoring data.^19^

In context of India, evidence on digitally enabling CHNWs with mHealth Applications is limited but promising. In Bihar, an intervention largely similar to ours and one that was a pilot-scale precursor to the intervention we evaluated found increased home visits by CHNWs to pregnant women and to mothers within the first week after birth by 11–12 percentage points (pp), breast feeding immediately after delivery by 12pp, and age-appropriate complementary feeding by 21pp.^20^ A quasi-experimental study used data from this RCT in Bihar along with data from two other state-level surveys to evaluate an audio-visual job-aid for CHNWs called Mobile Kunji implemented in eight districts in Bihar.^21^ The Mobile Kunji evaluation found consistent effects on birth preparedness, antenatal check-ups, exclusive breast feeding and complementary feeding across all three datasets. Another trial in Gujarat for a mobile app (ImTeCHO) used as a job aid for CHNWs found that home-visits by CHNWs during the first week of birth increased by 10.2pp, early initiation of breast feeding by 7.8pp, and exclusive breast feeding by 13.4pp.^22^ Results are awaited on the scaled-up version of ImTeCHO known as TECHO+but early qualitative evidence suggests that the uptake of mHealth intervention was hampered by poor technological literacy of older CHNWs.^23 24^ Kilkari is another large-scale digital intervention that involves health messaging directly to beneficiaries subscribed to receive such messages. Using system generated back-end data, authors found that reaching subscribers required multiple call attempts —up to 9 calls to reach 99.5% subscribers. Among those reached, 48% of the calls were listened to for at least half the duration of the content.^25^ We are aware of at least one more trial which is currently underway.^26^

Collectively, the existing evidence suggests that digital or mHealth applications have been effective as data capture, messaging, decision-support tools and job-aids for CHNW, and there is a possibility that higher order nutrition and health practices can be improved due to these interventions. However, it remains unknown whether these impacts are possible for at-scale digital health interventions integrated with national or regional health and nutrition programmes in LMICs.

This study addresses a critical gap in the evidence base by evaluating one of the largest digital health interventions for CHNWs in the world called Common Application Software (CAS) under the flagship nutrition programme, the Integrated Child Development Services (ICDS) in India. We evaluated the early effectiveness of ICDS-CAS in two states in India. We primarily aimed to assess whether the intervention improved CHNW services related to home visits and counselling of pregnant women and mothers of children <12 months. Additionally, as secondary outcomes, we evaluated the effectiveness of the digital health intervention on other nutrition services delivered by the CHNWs and infant and young child feeding practices.

### ICDS Program and the ICDS-CAS Intervention

The ICDS programme, launched in 1975, is a national flagship nutrition programme to support the health, nutrition and developmental needs of children below the age of six, and pregnant and lactating women, through a network of early childhood development and feeding centres called the Anganwadi Centres (AWCs) at the village-level.^27^ Each AWC is served by a CHNW called *Anganwadi* worker (AWW) who is a full-time government paid female worker—but her official position is that of a contractual staff and not permanent government staff—from the community. The ICDS programme serves an estimated 158 million beneficiaries through India’s 1.4 million CHNWs. Under the ICDS, CHNWs provide five core services: (1) supplementary food including hot-cooked meals and take-home rations (THR) (2) home visits to provide health and nutrition education to pregnant and lactating women on pregnancy care and infant and young child feeding practices; (3) growth monitoring for children; (4) preschool education for children 3–6 years of age; and (5) conducting a monthly event called the Village and Health and Nutrition Day for immunisation and other health-related services in coordination with the National Health Mission (NHM) frontline workers.

In 2012, the ICDS Systems Strengthening and Nutrition Improvement Programme (ISSNIP) was launched across 162 districts having high undernutrition burden. Under ISSNIP, a mobile app-based intervention was piloted for ICDS and NHM frontline workers to improve coordination and collective service delivery to pregnant women. In 2016, a modified mobile-app based intervention called CAS was rolled out for cadre of CHNWs under the ICDS by the Ministry of Woman and Child Development. At the time of this evaluation, ICDS-CAS intervention covered over 600,000 CHNWs from 347 districts across 28 states and more were likely to be covered.^28 29^

**Figure 1:**
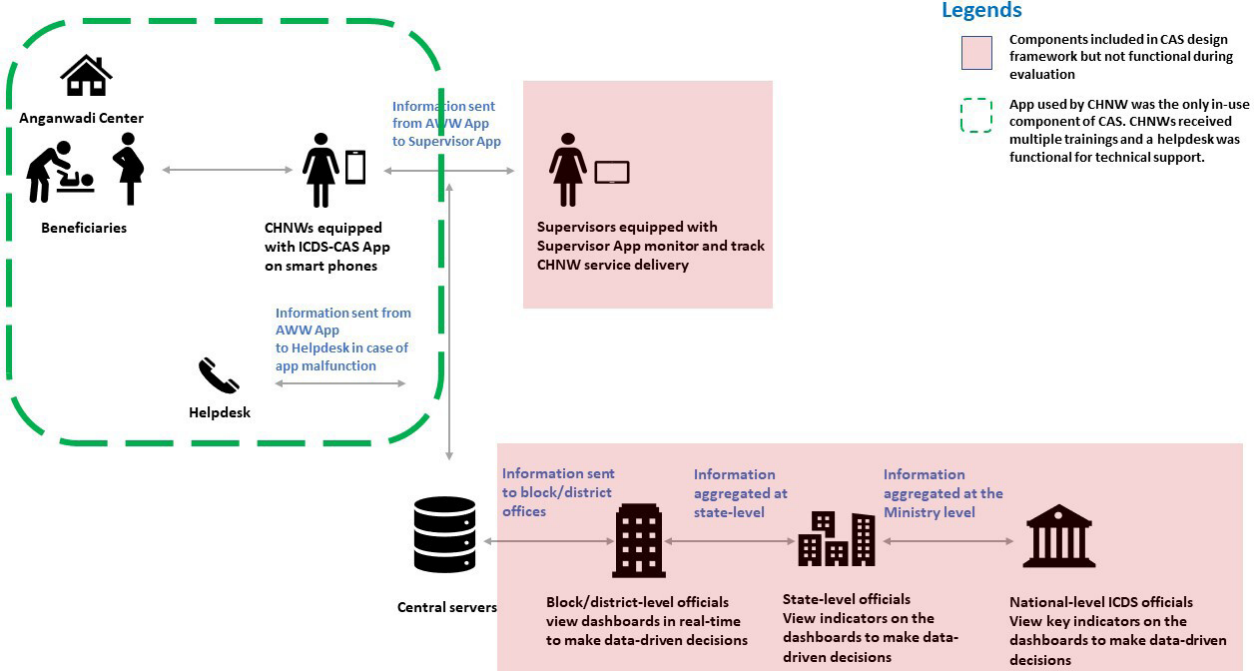
CAS design framework and functional component evaluated. CAS, Common Application Software; ICDS, Integrated Child Development Services.

ICDS-CAS sought to digitally enable CHNWs with a data capture module that digitised and replaced ten of the eleven paper registers maintained by the CHNWs to register and longitudinally track provision of services to different type of beneficiaries. ICDS-CAS also was designed to facilitate CHNW’s workflow management, remind her of upcoming home visits and services due to beneficiaries, provide checklists and a library of instructional videos as a job-aid during counselling, track growth status and immunisations for children, and report data for the programme’s monitoring. Figure 1 depicts the originally intended purpose and data flow for ICDS-CAS that was not only a CHNW level intervention but was also meant to support real time monitoring and decision making at all levels from the CHNWs’ immediate supervisors to the state-level ICDS director. ICDS-CAS intended to include three additional features : (1) a module for CHNWs to report supply chain constraints and other logistics issues; (2) a separate app for CHNW supervisors to monitor CHNWs remotely, assess quality of service delivery, and use as a job-aid to train CHNWs; and (3) web-based real-time dashboards for officials at the block, district, state and national levels to identify bottlenecks, prioritise local issues, and take data-driven decisions. But, the aforementioned features could not be implemented during our evaluation. Therefore, we evaluated the effect of digitally enabling CHNWs on the services they could deliver but not the effect of real-time monitoring or decision making features of the ICDS-CAS system. In 2020, Government of India discontinued CAS and replaced it with another system called the *Poshan* Tracker. It was beyond the scope of this impact evaluation to study why other components could not be implemented or the reasons why CAS was replaced with the *Poshan* Tracker.

**Figure 2:**
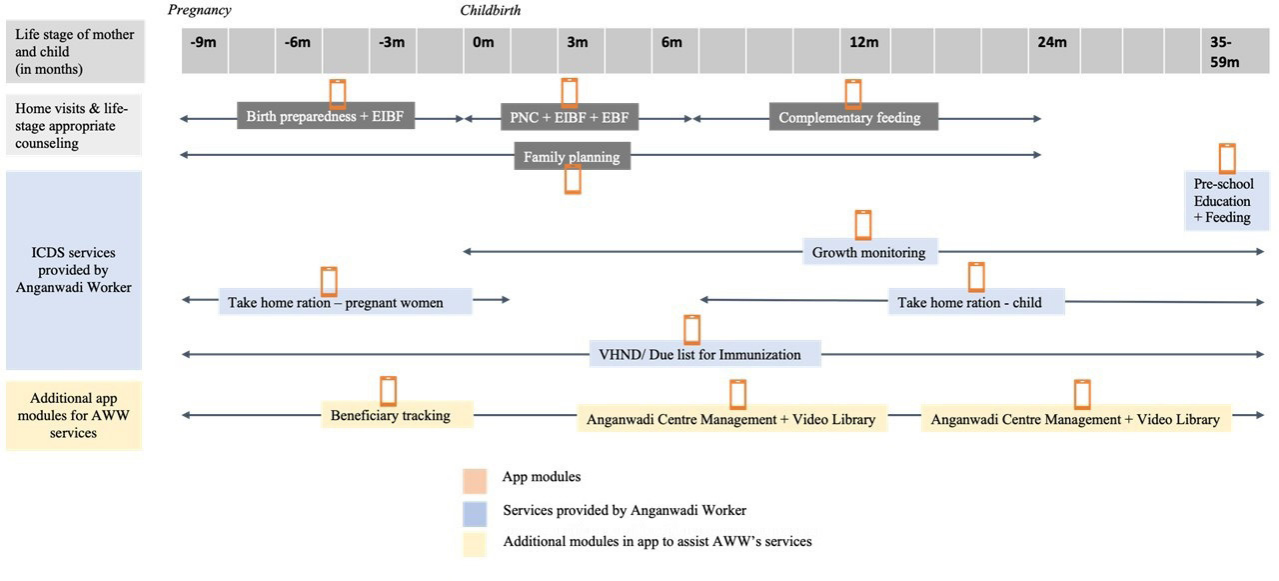
Mapping of ICDS services and CAP app modules. ICDS, Integrated Child Development Services; VHND, Village and Health and Nutrition Day; PNC, Post Natal Care; EIBF, Early Initiation of Breast Feeding; EBF, Exclusive Breast Feeding.

Figure 2 maps different ICDS services to different modules on the CHNW app to track and identify beneficiaries due for the service, provide the service and update the records for longitudinal tracking. All CHNWs were provided training over multiple rounds on using the CHNW app. Helpdesks at block and district levels were available for technical support.

Based on the WHO classification of digital health intervention,^30^ this intervention would broadly fit the following classifications: (2.1) client identification and registration; (2.2) client health records; (2.3) healthcare provider decision support; (2.7) health worker activity planning and scheduling; (1.1) targeted client communication (during home visits) and (4.1) data collection, management and use.

## Methods

### Theory of change

**Figure 3:**
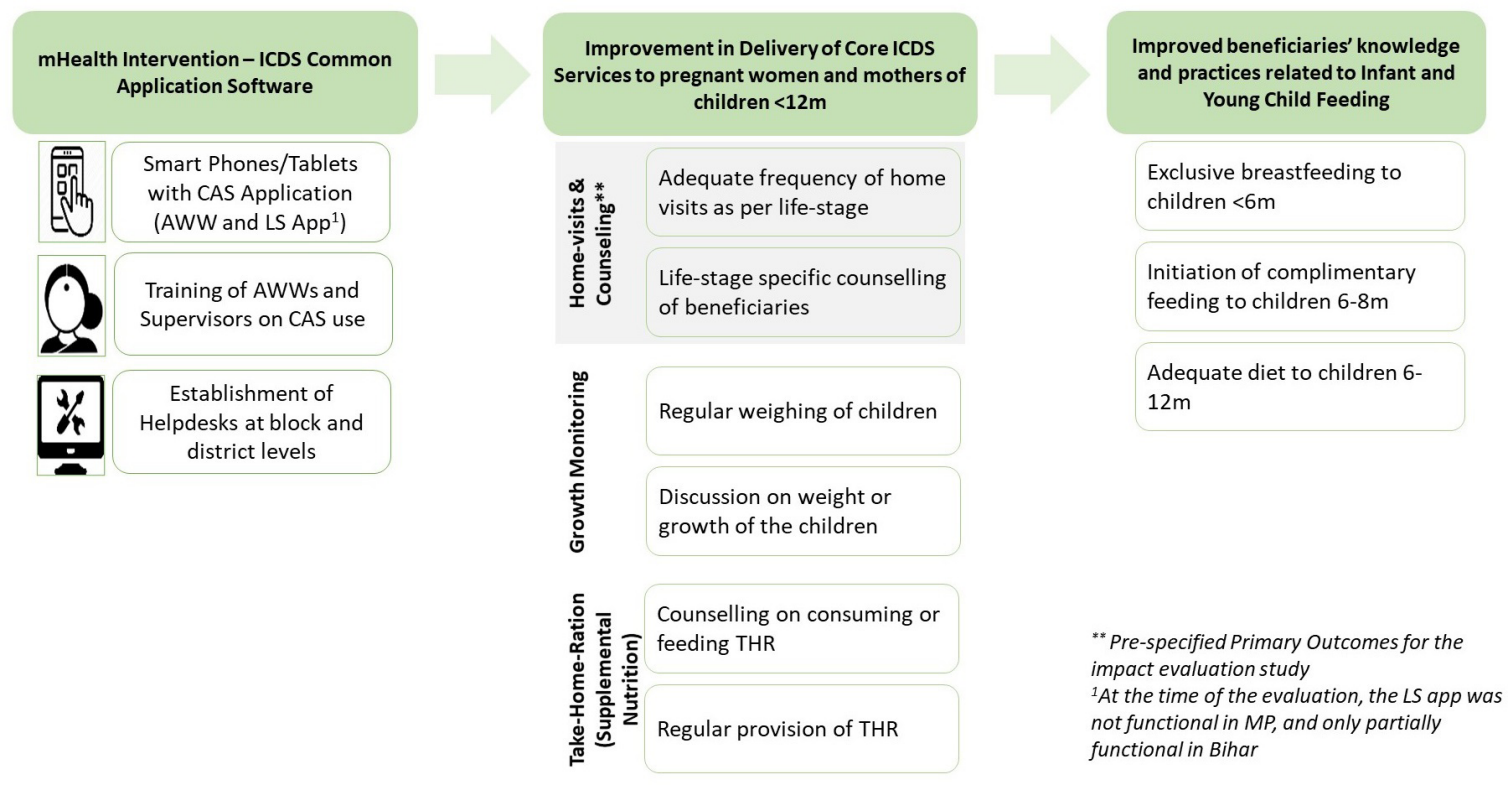
Theory of change of digitally enabling CHNWs with CAS. AWW, Anganwadi worker; CAS, Common Application Software; ICDS, Integrated Child Development Services; MP, Madhya Pradesh; THR, take-home ration.

Figure 3 presents the hypothesised theory of change we evaluated. The CAS app for CHNWs was expected to improve CHNW service delivery related to home visits and counselling as per ICDS guidelines specific to the life stages (trimester of pregnancy, postpartum period and age of child), growth monitoring consisting of weighing of the child and discussion of growth with the mothers, and counselling and provision of THR to pregnant women and mothers of children <12 months. We further hypothesised that improvement in these ICDS services can improve infant and young child feeding practices.

### Study design

We used a village-matched, quasi-experimental design with repeated cross-sectional preintervention and postintervention measurements to assess the impact of the digital health intervention. The impact parameter of interest is intention-to-treat effect conditional on matching and sample restriction. All primary and secondary outcomes were measured based on interviews with pregnant women and mothers, and the community-level covariates were based on CHNWs’ interviews. Study methods, details of matching and rationale have been published previously.^31^ Reporting in this article follows the STROBE (STrengthening the Reporting of OBservational studies in Epidemiology) guidelines for quasi-experimental evaluation studies.^32^

### Patient and public involvement

Patients or the public were not directly involved in the design, or conduct, or publication, or dissemination plans of our research. However, the research design was presented to government departments, funders, implementing partners of the intervention including non-governmental organizations (NGOs) to build their support for the study. Periodic updates on study progress were given to these stakeholders, and results were presented to them as well.

### Study setting and timelines

The staggered rollout of the digital health intervention started in October 2016 across several districts and states under the larger umbrella of a system strengthening project for nutrition (https://projects.worldbank.org/en/projects-operations/project-detail/P121731). The evaluation was conducted in two purposively selected states, Madhya Pradesh (MP) and Bihar, in discussion with the Ministry of Woman and Child Development and the funding agency. The states differed in prevailing health and nutrition issues as well as the capacity of the state health and nutrition systems. Bihar has a higher burden of undernutrition than MP—49.3% of children <5 years in Bihar and 43.6% in MP were stunted and 63% of mothers in MP and 49% in Bihar reported that their children <6 years received some service from the AWCs.^33^ The under-5 mortality is high in both states at 65 and 58 per 1000 live births in MP and Bihar, respectively. Only 34% of infants in Bihar and MP were fed within the first hour of birth, and exclusive breast feeding at 6 months was 53% and 58%, respectively. Even more alarmingly, only 8% in Bihar and 7% in MP of children 6–23 months received an adequate diet.

**Figure 4:**
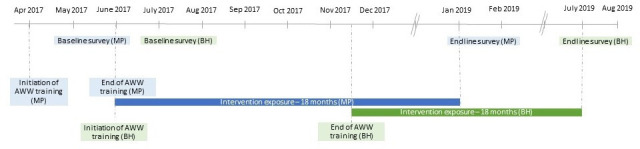
Timeline of the intervention and evaluation surveys. AWW, Anganwadi worker; BH, Bihar; MP, Madhya Pradesh.

Data were collected at two time points, before the launch of the full intervention (May–August 2017), and at follow-up (2019: January–February in MP and July–August in Bihar). The intervention and survey timelines are shown in figure 4. Prior to the baseline survey in May–August 2017, the CHNWs in the intervention districts in MP and Bihar had received smartphones, installed the app and received first of the four rounds of training on the app use. However, the full set of four trainings were concluded and the app usage started by July (MP) and December (Bihar), 2017 after the baseline data collection. The endline survey in Bihar was postponed by almost 6 months to allow ICDS services to normalise after a month-long CHNW strike in February–March 2019.

### Statistical methods

We estimated intention-to-treat impacts of the intervention as pre-specified in the study protocol, separately for MP and Bihar, using two models.

$$Model1:Y_{ij,t=1}=\beta_{0}+\beta_{1}.T_{j}+\beta_{2}.{\overset{-}{Y}}_{j,t=0}+PairID_{K}+\varepsilon$$

$$Model2:Y_{ij,t=1}=\beta_{0}+\beta_{1}.T_{j}+\beta_{2}.{\overset{-}{Y}}_{j,t=0}+\beta_{3}.P_{j}+PairID_{D}+\;\beta .Z+\varepsilon$$

where, $Y_{ij,t=1}$ is the outcome of interest for beneficiary *i* in village *j* at endline (*t=1*); $T_{j}$ is an indicator variable f denoting the treatment status of the digital health intervention; ${\overset{-}{Y}}_{j,t=0}$ is the village-level average of outcome Y at baseline (*t=0*); $PairID_{K}$ and $PairID_{D}$ are fixed effects for *k* pairs of matched villages or *d* pairs of matched districts; *P_j_* is the propensity score estimated in the baseline; Z is a set of 20 covariates identified based on balance test results to control for observed imbalances and to improve precision; ε is the model error term; and $\beta_{1}$ is the estimated risk difference or the effect of the intervention on outcome Y. Note, the intervention was assigned at the district level so that all villages in a district are in either intervention or comparison group. The pairs of intervention-comparison villages were matched using nearest neighbour propensity score matching method as described in the protocol.^31^

Preintervention balance was assessed using model 1 except the term ${\overset{-}{Y}}_{j,t=0}$ for a range of CHNW, household and beneficiary characteristics.

SEs in model (1) were clustered at the village level given village-pair fixed effects were specified, and standard errors in model (2) were clustered at the village-pair level to account for village pairing. Given the evidence of some imbalance between the intervention groups, results from model (2) are mainly discussed in this paper.

All analyses were done in STATA V.15, documented in a DO file, verified for code consistency (by AP and LG), and replicated by two different analysts (SN and SRP).

### Sampling

**Figure 5:**
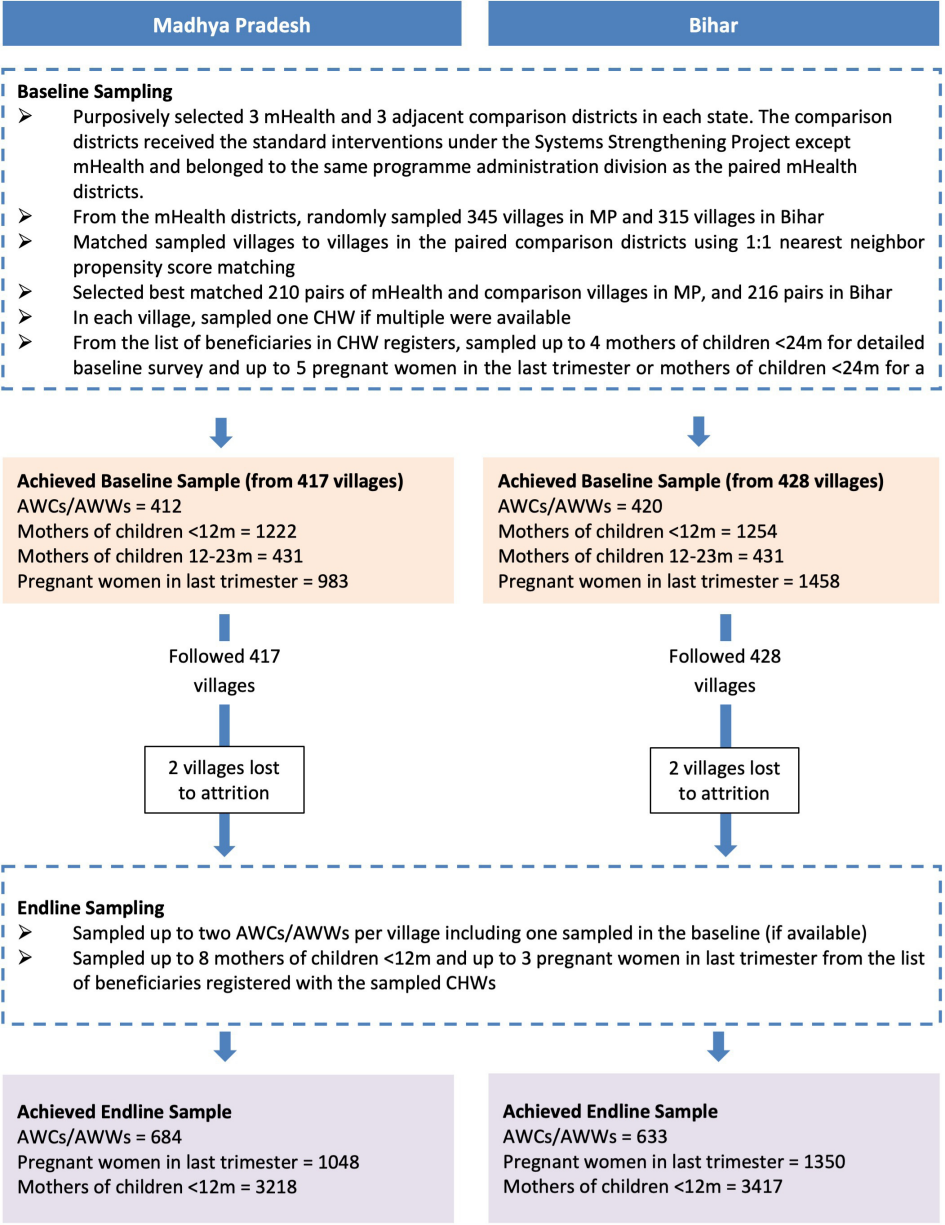
Sampling of study participants. AWCs, Anganwadi centres; AWWs, Anganwadi workers; CHW, Community Health Workers also called CHNW in the paper.

Sample design and sampling procedures are published in the protocol^31^ and updated in figure 5. The sample was powered to detect a difference of 5–9pp from the counterfactual levels between 10% and 50% with intracluster correlation coefficient between 0.15 and 0.30 assuming 1200 respondents from 200 villages in each arm in each state.

The achieved sample consisted of 210 pairs of villages in MP and 216 pairs in Bihar matched using 1:1 nearest neighbour propensity score matching with Census 2011 village level characteristics from three pairs of CAS and non-CAS ISSNIP districts which were purposively selected in MP and Bihar. Baseline surveys were completed in 417 (out of 420) villages in MP and 428 (out of 432) villages in Bihar. At the endline, 4 out of 845 villages were lost to follow-up and this minor loss is assumed as random. The endline sample in MP was 684 CHNWs from 415 villages, 1048 pregnant women in last trimester and 3218 mothers of children <12 months. The endline sample in Bihar was 633 CHNWs from 426 villages, 1350 pregnant women in last trimester, and 3417 mothers of children <12 months. This study excluded women not registered with the CHNWs and mothers of children 12–59 months who were covered by the ICDS-CAS intervention, because the primary outcomes would be most relevant and sensitive for pregnant women and mothers with children <12 months given the exposure period of 16–18 months.

### Outcomes and their measurement

The primary outcome indicators were constructed using aided recall by beneficiaries of home visits and counselling received from CHNWs during 3 months prior to the survey as prespecified in the study protocol. ICDS programme guidelines specify the number of home visits and counselling messages a beneficiary should receive at different life stages (ranging from the second trimester of pregnancy up to the child completing 24 months of age). Accordingly, the outcome indicator for adequate number of home visits was set to 1 if the respondent reported receiving the minimum number of home visits in the last 3 months, and 0 otherwise. The outcome indicator for life-stage appropriate counselling was set to 1 if the respondent recalls receiving at least half the counselling messages appropriate for their life stage as per ICDS guidelines in the past 3 months, and 0 otherwise.

Secondary outcomes included exposure to other nutrition programme services, such as food supplements and growth monitoring, as well as infant and young child feeding practices. Online supplemental table S1 describes in detail the construction of primary and secondary outcome indicators and the covariates used in the analyses, except those used only to test for preintervention balance.

10.1136/bmjgh-2021-007298.supp1 Supplementary data

### Role of the funding source

The funder provided input to the overall evaluation design along with other partners. The funder was not involved in data collection, data analysis, data interpretation or writing of the manuscript. Funders reviewed the draft manuscript, but all final decisions rested with the authors. The corresponding author had full access to all the data and had final responsibility for the decision to submit for publication.

## Results

### Sample characteristics and balance

Table 1 summarises sample characteristics at endline in the intervention and comparison groups in MP and Bihar. Compared with Bihar, MP had more pucca constructed AWCs (84% vs 54%), more centres with drinking water (65% vs 47%) and toilets (42% vs 18%), a larger proportion of CHNWs fully trained (68% vs 46%) and fewer CHNWs reporting problems in supply of supplementary nutrition or THR (11% vs 45%). A higher proportion of households in MP belonged to scheduled castes, had built a toilet and owned agricultural land.

**Table 1:** Characteristics of survey respondents and exposure of community health workers to CAS, endline survey

Variables	MP				Bihar			
	Comparison		Intervention		Comparison		Intervention	
	N	Mean (SD)*	N	Mean (SD)*	N	Mean (SD)*	N	Mean (SD)*
Individual characteristics†								
Beneficiary’s age (in completed years)	2021	24.0 (3.86)	2245	23.9 (3.61)	2384	24.3 (4.27)	2383	24.7 (4.65)
Beneficiary’s education (in years)	2021	5.3 (4.43)	2245	5.5 (4.28)	2382	4.5 (4.81)	2349	4.0 (4.67)
Beneficiary works to earn income	2021	25.7%	2245	25.4%	2384	8.8%	2383	11.1%
Beneficiary finds it easy to reach the AWC	2021	83.1%	2245	81.7%	2384	76.4%	2383	79.1%
Beneficiary has an Aadhar card	2021	97.8%	2245	97.3%	2384	96.6%	2383	96.4%
Beneficiary owns a bank account	2021	90.9%	2245	90.1%	2384	84.1%	2383	87.0%
Number of pregnancies in beneficiary’s lifetime	2021	2.4 (1.50)	2245	2.3 (1.40)	2384	2.7 (1.64)	2383	2.9 (1.73)
Household Characteristics†								
Household belongs to scheduled castes/tribes	2021	55.9%	2245	51.1%	2384	36.0%	2383	36.6%
House is of pucca construction	2021	33.2%	2245	38.0%	2384	52.7%	2383	46.5%
No of rooms used for sleeping in the house	2021	2.1 (1.00)	2245	2.1 (1.09)	2384	2.2 (1.31)	2383	2.1 (1.30)
Household owns agricultural land	2021	74.0%	2245	70.7%	2384	42.8%	2383	40.5%
House has a functional toilet	2021	73.8%	2245	61.9%	2384	36.9%	2383	47.9%
CHNW Characteristics‡								
CHNW’s age	353	38.1 (8.71)	331	39.7 (9.15)	294	39.3 (8.93)	339	37.7 (7.03)
CHNW’s education (in years)	353	10.6 (3.78)	331	10.9 (3.47)	294	11.5 (2.1)	339	11.8 (2.03)
CHNW belongs to a schedules caste/tribe	353	58.6%	331	47.7%	294	15.0%	339	20.1%
Number of years of experience as CHNW	353	14.3 (7.97)	331	15.7 (8.58)	294	15.6 (9.22)	339	12.2 (5.04)
CHNW owns a smartphone	353	32.6%	331	100.0%	294	59.2%	339	94.7%
CHNW is trained in seven topics under ISSNIP	353	75.4%	331	74.6%	294	53.1%	339	51.9%
CHNW has frequent interactions with LS	353	49.9%	331	53.5%	294	60.5%	339	54.6%
CHNW reported problems with THR supply	353	12.5%	331	7.3%	294	35.7%	339	53.4%
Characteristics of AWC‡§								
Population covered by the AWC	310	698.1	303	829.1	222	1081.8	312	1024.0
AWC has pucca construction	353	80.5%	331	90.6%	294	58.2%	339	49.9%
AWC has drinking water on premises	352	65.9%	329	67.8%	288	49.3%	323	49.5%
AWC has toilet on premises	352	52.8%	329	48.6%	288	18.4%	323	24.2%
AWC has functional child weighing scale	353	84.4%	331	83.4%	294	75.9%	339	83.5%
AWC has growth charts for children	353	45.6%	331	50.2%	294	38.4%	339	35.4%
Exposure of CHNWs to ICDS-CAS Intervention‡								
Received training on using App			331	97.9%			339	97.9%
CHNWs own a smart phone			331	100.0%			339	94.7%
CHNWs who reported carrying their phone during home visits in the last 30 days			331	99.1%			339	88.2%
CHNWs who reported that they always showed the App content/messages to beneficiaries during home visits in the last 30 days			331	68.6%			339	69.9%

*For continuous variables mean and SD are reported. For binary variables percentage is reported.

†Based on self-report from beneficiary surveys.

‡Based on self-report from CHNW surveys.

§Infrastructure at AWC was assessed as per the observations by enumerators.

AWC, Anganwadi centre; CAS, Common Application Software; CHNW, community health and nutrition worker; ICDS, Integrated Child Development Service; ISSNIP, ICDS Systems Strengthening and Nutrition Improvement Programme.

As reported in online supplemental table S2, groups were well balanced at the baseline on all primary outcomes and most beneficiary characteristics, but there were some differences in CHNW characteristics, service delivery and secondary outcomes related to growth monitoring and complementary feeding. Even at the endline, there were some important differences between the intervention and comparison groups in CHNW and beneficiary characteristics and practices which could not have been influenced by the intervention (online supplemental table S3).

Given evidence of imbalance in some of the preintervention covariates and differences at the endline in two group, results from model 2 are mainly discussed in this paper. We also flag and discuss results where the magnitude of the effect estimated by models 1 and 2 are ‘qualitatively’ different; often they agree with each other.

### Exposure to the intervention

As prespecified in the protocol,^31^ we estimated the intention-to-treat impact parameter which assumes that all CHNWs and beneficiaries were exposed to the intervention as per the original assignment. Nearly 98% of the CHNWs in the intervention districts in both states received the training, 100% in MP and 95% in Bihar reported owning a smart phone, and 99% in MP and 88% in Bihar carried the smart phone with app during home visits as reported in table 1. Close to 70% of the CHNWs reported ‘always’ using the app content during home visits.

### Primary outcomes

At endline, mothers in the intervention group were more likely to receive the appropriate number of home visits in both states (MP: comparison mean=42%, effect=8 pp (95% CI 4pp to 13pp; p<0.001), Bihar: comparison mean=24%, effect=8 pp (95% CI 4pp to 12pp; p<0.001)) (table 2). Mothers were also more likely to recall at least half of the counselling messages that they were meant to receive (MP: comparison mean=28%, effect=12 pp (95% CI 7pp to 16pp; p<0.001). Bihar: comparison mean=9%, effect=8 pp (95% CI 5pp to 11pp; p<0.001)). The magnitude of the effect size is practically the same between models 1 and 2 suggesting lack of confounding bias, and thus, attributable impacts.

**Table 2:** Effect of CAS on primary outcomes: home visits and counselling to mothers and pregnant women

	MP		Bihar	
	Model 1	Model 2	Model 1	Model 2
**Mothers of children <12 months (n=6635)**				
% of mothers who received adequate number of home visits in last 3 months				
Beta	0.06 (0.03–0.10); p<0.001	0.08 (0.04–0.13); p<0.001	0.07 (0.04–0.10); p<0.001	0.08 (0.04–0.12); p<0.001
Comparison mean	0.42	0.42	0.24	0.24
% of mothers who received at least 50% of the correct counselling messages as per their life stage				
Beta	0.11 (0.08–0.14); p<0.001	0.12 (0.07–0.16); p<0.001	0.08 (0.06–0.10); p<0.001	0.08 (0.05–0.11); p<0.001
Comparison mean	0.28	0.28	0.09	0.09
**Pregnant women in third trimester (n=2398)**				
% of pregnant women who received adequate no of home visits in the third trimester				
Beta	0.02 (−0.03 to 0.08); p=0.41	0.03 (−0.04 to 0.11); p=0.35	0.02 (−0.03 to 0.07); p=0.35	0.03 (−0.03 to 0.09); p=0.33
Comparison mean	0.55	0.55	0.27	0.26
% of pregnant women who received at least 50% of the correct counselling messages as per their life stage				
Beta	0.09 (0.04–0.15); p<0.001	0.09 (0.02–0.16); p=0.01	0.05 (0.00–0.10); p=0.04	0.03 (−0.02 to 0.09); p=0.25
Comparison Mean	0.43	0.43	0.24	0.23

CAS, Common Application Software.

The proportion of pregnant women receiving adequate number of home visits was not different between the groups at the endline (MP: comparison mean=55%, effect=3 pp (95% CI −4pp to 11pp; p=0.353), Bihar: comparison mean=26%, effect=3 pp (95% CI −3pp to 9pp; p=0.327)). However, the recall of at least half of the life-stage appropriate messages by pregnant women in the intervention group was higher in MP (MP: comparison mean=43%, effect=9 pp (95% CI 2pp to 16pp; p=0.009)).

### Secondary outcomes

In Bihar, the proportion of mothers reporting weighing of their child or reporting that the CHNW discussed child’s weight/growth more than doubled in the intervention group, with little difference in the magnitude of the effect between models 1 and 2 (table 3). In MP, the CAS intervention did not result in any difference between groups at endline. There was no effect of CAS on receipt of THR at least once a month by mothers. However, in both states 8%–10% more mothers in the intervention group reported receiving counselling on consumption of THR.

**Table 3:** Effect of CAS on secondary outcomes: growth monitoring and supplementary nutrition services by community health workers

	MP		Bihar	
	Model 1	Model 2	Model 1	Model 2
**Growth monitoring of infants <12 months (n=6635)**				
% of mothers who reported that their child aged 0–12 months was weighed by CHNW at least once in last 3 months				
Beta	0.00 (−0.03 to 0.04); p=0.87	0.01 (−0.03 to 0.05); p=0.66	0.22 (0.18–0.25); p<0.001	0.24 (0.19–0.28); p<0.001
Comparison mean	0.66	0.66	0.18	0.17
% of mothers who reported that CHNW discussed their child’s weight or showed child’s growth chart				
Beta	−0.02 (−0.05 to 0.01); p=0.20	−0.00 (−0.05 to 0.04); p=0.86	0.15 (0.13–0.18); p<0.001	0.15 (0.13–0.20); p<0.001
Comparison mean	0.48	0.48	0.15	0.15
**THR supplementation to mothers of children <12 months (n=6635)**				
% of mothers (0–12 months child) who received THR at least once a month from CHNW				
Beta	−0.01 (−0.04 to 0.01); p=0.34	−0.00 (−0.04 to 0.04); p=0.86	−0.04 (−0.07 to 0.00); p=0.046	−0.03 (−0.08 to 0.02); p=0.24
Comparison mean	0.72	0.72	0.35	0.34
% of mothers (0–12 months child) who recall being counselled about mother’s/child’s THR consumption by CHNW in last 3 months				
Beta	0.09 (0.06–0.12); p<0.001	0.10 (0.05–0.14); p<0.001	0.08 (0.06–0.10); p<0.001	0.09 (0.06–0.12); p<0.001
Comparison mean	0.39	0.39	0.15	0.14

CHNW, community health and nutrition worker; THR, take-home ration.

In both states, a higher proportion of mothers in the intervention areas recalled being counselled by the CHNW about exclusive breast feeding, timely initiation of complementary feeding, diet diversity, and adequate frequency of meals for children >6 months of age as was intended under the theory of change for CAS (table 4). However, no impacts were observed on infant and young child feeding practices.

**Table 4:** Effect of CAS on secondary outcomes: exposure to counselling and child feeding practices

	MP		Bihar	
	Model 1	Model 2	Model 1	Model 2
**Exclusive breast feeding to children <6 months (n=3490)**				
% of mothers who recalled being counselled about exclusive breast feeding by CHNW				
Beta	0.12 (0.08–0.16); p<0.001	0.11 (0.06–0.17); p<0.001	0.11 (0.06–0.16); p<0.001	0.11 (0.05–0.18); p<0.001
Comparison mean	0.61	0.61	0.35	0.34
% of children who were only fed breastmilk during the previous 24 hours				
Beta	0.02 (−0.01 to 0.05); p=0.19	0.02 (−0.02 to 0.07); p=0.26	−0.09 (−0.13 to −0.05); p<0.001	−0.07 (−0.12 to −0.02); p=0.01
Comparison mean	0.81	0.82	0.49	0.49
**Timely initiation of complementary feeding to children 6–8 months (n=1719)**				
% of mothers who recalled being counselled about the right time to start complementary feeding by CHNW				
Beta	0.10 (0.02–0.17); p=0.015	0.10 (0.02–0.18); =0.02	0.09 (0.03–0.15); p=0.004	0.13 (0.06–0.20); p<0.001
Comparison mean	0.46	0.46	0.17	0.17
% of children who received solid, semisolid, or soft foods during previous 24 hours				
Beta	−0.04 (−0.11 to 0.04); p=0.34	−0.00 (−0.08 to 0.07); p=0.95	0.01 (−0.04 to 0.07); p=0.57	−0.01 (−0.07 to 0.06); p=0.86
Comparison mean	0.71	0.71	0.67	0.68
**Minimum dietary diversity and adequate frequency of meals to children 6–11 months (n=3145)**				
% of mothers who recalled being counselled about dietary diversity and adequate frequency of meals for their child by CHNW				
Beta	0.08 (0.03–0.13); p=0.003	0.11 (0.05–0.17); p<0.001	0.10 (0.06–0.14);p<0.001	0.11 (0.06–0.15); p<0.001
Comparison Mean	0.44	0.44	0.17	0.17
% of children who received adequate diet as per their age during previous 24 hours				
Beta	0.02 (-0.01–0.04); p=0.27	0.02 (-0.01–0.06); p=0.21	0.01 (-0.02–0.04); p=0.40	0.01 (-0.02–0.05); p=0.56
Comparison Mean	0.07	0.07	0.12	0.12

CAS, Common Application Software; CHNW, community health and nutrition worker.

## Discussion

To our knowledge, this is the first study to provide robust evidence of an at-scale digital health intervention that enabled CHNWs with a data capture, decision support and job-aid mobile application working in a national nutrition programme in two Indian states with different population and programme contexts. This digital health intervention resulted in higher home visits and life-stage specific counselling to mothers of children <12 months, both primary outcomes for this evaluation.

Although the absolute magnitude of this effect was similar in both Bihar and MP, the relative effects were larger in Bihar, perhaps due to poorer baseline levels of service delivery than that in MP. Our study did not find any impact on adequate number of home visits to pregnant women in Bihar or MP, but a higher proportion of pregnant women in MP received more effective counselling. One possible explanation is that digitally enabling CHNWs is most useful when service delivery guidelines are complex and change substantially as per the life-stage of the beneficiaries. This was the case for mothers of children <12 months, but not for pregnant women who required only one visit any time in the last trimester as per ICDS guidelines which, arguably, can be remembered and pursued by CHNWs even without the CAS App.

Growth monitoring and provision of supplementary nutrition (THR) are among the core nutrition services provided by CHNWs. The stronger impacts on growth monitoring—weighing of children and discussion of the child’s growth with mothers—that we found in Bihar, compared with MP, likely reflect the diminishing returns of ‘enabling through digital technology’ when the service levels are already high as was the case in MP where growth monitoring was more prevalent even at baseline. The finding that more mothers in the intervention group reported receiving counselling on consumption of THR, but that there were no impacts on actual receipt of THR likely reflect that the digital empowerment of CHNWs affected those services that were within the immediate control of the CHNW (*i.e.*, counselling), but not those outside the CHNW’s direct control such as THR provision which is controlled by supply chains at state and district levels. A similar lack of effect was also noted in the evaluation of Mobile Kunji intervention for contraceptives and vaccines use because of the affected supply chains.^21^

Finally, no impacts were observed for the higher order outcomes of infant and young child feeding behaviours, which is expected because practices are not only driven by counselling and knowledge, but also agency, enabling environment, and resources available to the women and households.

### Comparison with other digitally enabled CHNW evaluations

The magnitude of effects on appropriately timed home visits are somewhat comparable with results from randomised controlled trials of similar digital interventions for community health workers at pilot-scale. Compared with our finding of 8pp effect on homevisits to mothers, a trial in Bihar had found an impact of 12pp^20^ and another trial in Gujarat had found an impact of 10.2pp on visits during the first week after the birth of the child.^22^ However, our study did not find any impact on visits to pregnant women unlike the above pilot trials in Bihar and Gujarat. It is possible that the behaviour change tools and approaches used in CAS application were not as effective as the tools and approaches used in the pilot programmes. For example, Mobile Kunji intervention that focused on human-centric audio-video and interactive voice response messaging in eight districts of Bihar was strongly associated with a range of behavioural and nutrition outcomes.^21^

Across all four of the infant and young child feeding counselling areas—from early initiation of breast feeding to complementary feeding—we found that more mothers in the intervention group recalled receiving messages on recommended practices. This is promising and suggests that digital technologies can enable provision of more age-appropriate content of counselling. However, our study did not find impacts on infant and child feeding practices, unlike the pilot-scale trials.^20 22^ It is likely that these results highlight the challenges in replicating success of small, well-controlled, and intensive pilots in large-scale real-life programmes. In this respect, our findings are similar to an observational study of a national-scale SMS-based data reporting and health monitoring system in Rwanda which found no impact of the at-scale intervention on antenatal care visits, institutional deliveries, postnatal care visits, and malnutrition screening.^19^ It is also possible that despite the delivery of appropriate counselling content, other programme components that support nutrition behaviour change may have been limited. For example, other large-scale behaviour change programmes invested heavily in shaping other determinants such as community norms or engaged influential members such as fathers and grandmothers.^34^ It is also possible that other non-knowledge constraints to behaviour change—such as maternal time, workload, or household resources—were not fully addressed.

### Limitations

Our study is not without limitations in identifying unbiased estimates of the programme effects. Due to the nature of the rollout, we could not design a randomised controlled trial that would have been an ideal design to prove causality. We relied on an observational design with the matching procedure that prioritises reducing selection bias or confounding but at the cost of representativeness of the sample. Therefore, the results are conditional on the matched sample which excludes villages not on common support of propensity score.^31^

Our village-level matching and use of a repeated cross-sectional design can theoretically ensure control of time invariant village-level confounders but not beneficiary-level or time-variant village-level confounders. However, the strong agreement between the results with and without controlling for household, individual and village-level covariates suggests that the bias due to imbalanced confounders was minimal.

We also mainly rely on recall by survey participants in constructing our indicators and not on any objective measurements or data. We did not have access to backend data from the CAS app or ICDS system which could have provided us with more objective measurements. However, even if we had these data, per the intervention’s design, there would have been a differential bias in measurements—the monthly progress data in the intervention group would have been more complete, timely, and less error prone because of CAS app, while the comparison group would have used the paper-based registers to compile such data which is a completely different system and cannot be compared with CAS. Although the recall biased indicators can have measurement errors, the responses of beneficiaries in the intervention and control groups should not be differentially biased due to John-Henry or Hawthorne biases because, arguably, the only difference beneficiaries could perceive was seeing a mobile phone in the hands of a CHNW. On other hand, access to the system-generated data on CHNW engagement with the app could have allowed us to perform heterogeneity analysis by CHNW performance and engagement with the app and to study if that lead to improved outcomes for beneficiaries.

## Conclusion

Overall, our study provides generalisable evidence on how much value digitally enabling CHNWs can add when such technology is integrated with an at-scale health programme. Our study highlights that health workers can and do use technology and their service delivery can be improved to the extent these services are under their control and not dependent on system-level constraints such as supply chain and/or infrastructure. We also find that not all services can be improved beyond a certain level because of the diminishing returns when the service levels are already high as was the case in MP for growth monitoring. Collectively, the improvements in CHNW performance may not translate into higher order behaviour change or health and nutrition outcomes, and that it delivers impacts that are well within the theory of change of the intervention.

It can be argued that larger impacts on higher-order outcomes may have been possible had ICDS-CAS been implemented with all components including real-time monitoring and decision support and for a longer duration. Indeed, certain intended components of the digital intervention were not functional such as module to report on THR supply-chain issues, app for the CHNW supervisors, and web-enabled dashboard for ICDS officials. Further, our evaluation states did not have access to the system data from CAS or were able to link CAS with their own state-level monitoring systems. Perhaps, these lacunae compromised the data-driven decision-making and programme management. However, more certainly, presence of such larger systemic bottlenecks merely reflects the challenges digital intervention will face when they are integrated with national-scale programmes in LMICs such as India.

Overall, digital health interventions can neither substitute efforts required to strengthen nutrition and health systems nor address structural barriers to achieve health and nutrition behavioural improvements. Current evidence on at-scale digital health interventions in LMICs—including ours—is mainly about service delivery improvements. Therefore, future evaluations should focus on studying whether and how higher-order impacts including infant and young child feeding practices can be delivered with use of digital health interventions at-scale. For example, future research can explore whether digital interventions can instil greater accountability and efficiency across the entire programme organisation in addition to CHNWs, and whether it can address bottlenecks such as supply of commodities/rations. More research is also required to understand how nutrition programmes can overcome structural barriers at a household and community-level and then consider how such digital technology can be leveraged to further enable service delivery to address these barriers. Backend data generated by digital health applications can and should also be made available to researchers and policy makers for more insightful operations research. Finally, promising digital health interventions can be subjected to rigorous technoeconomic feasibility and cost-benefits analyses to justify the investment and ensure sustainability prior to their scale-up.

## Data Availability

Data are available on reasonable request. Anonymised data used in this study can be requested from the corresponding author. The authors also intend to make the data publicly available as per the funding agency’s policies and procedures at a later date.
