# Supplementary material for: Can digitally enabling community health and nutrition workers improve services delivery to pregnant women and mothers of infants? Quasi-experimental evidence from a national-scale nutrition programme in India
Source: BMJ Glob Health. 2022 Jul 14;6(Suppl 5):e007298. doi: 10.1136/bmjgh-2021-007298 (PMC9296874; doi:10.1136/bmjgh-2021-007298)
Supplement: Supplementary data [file bmjgh-2021-007298supp001.pdf]

Table S1. Construction of Primary and Secondary Outcome Indicators and other Covariates

Name	Description	Type	Notes on Construction and Measurement
**Outcomes**			
**Primary Outcomes at Beneficiary Level**			
adeq_num_hv_3m	Received adequate number of home visits in last three months (mother)	Binary	ICDS provides detailed guidelines specifying the number of home visits a beneficiary should receive at different life stages. For example, a mother must receive at least one home visit per month when the child is between 6-8 months of age. The indicator is coded 1 if the mother received adequate number of visits in the last three months, and 0 otherwise. We use an additional assumption that every mother should receive at least one home visit in a given 3-month period.
recd_50pct_messages	Received at least half the life-state appropriate counselling messages (mother)	Binary	ICDS provides information about the various life-stage appropriate counselling messages that should be provided to mothers at different life stages. The indicator is coded 1 if the mother received at least half the appropriate counseling messages in the last three months, and 0 otherwise.
adeq_num_hv_3m_PW	Received adequate number of home visits in last three months (pregnant woman)	Binary	ICDS provides detailed guidelines specifying the number of home visits a beneficiary should receive at different life stages. For example, a pregnant woman must receive at least two home visits in the last trimester, preferably in the 7th and 9th months. The indicator is coded 1 if the pregnant woman received one or more home visits in her last trimester, and 0 otherwise.
recd_50pct_mess_PW	Received at least half the life-state appropriate counselling messages (pregnant woman)	Binary	ICDS provides information about the various life-stage appropriate counselling messages that should be provided to pregnant women in the last trimester. The indicator is coded 1 if the pregnant women received at least half the appropriate counseling messages in the last three months, and 0 otherwise.
**Secondary Outcomes at Beneficiary Level (Other services by the Community Health Worker [CHW])**			
weighed3months	Child was weighed by CHW at least once in last three months	Binary	Coded 1 if the mother reported that the CHW weighed her child at least once in the last three months, and 0 otherwise.
discuss_childweight	CHW discussed child's weight with mother in last three months	Binary	Coded 1 if the mother reported that the CHW discussed her child's weight or nutritonal status in the last three months, and 0 otherwise.
recd_THR_reg	Receives THR for herself/child one or more times a month from AWC	Binary	Coded 1 if the mother reported that she receives THR one or more times a month from the AWC, and 0 otherwise.
counsel_THR	CHW counselled mother on THR consumption for mother/child	Binary	Coded 1 if the mother reported that she was counseled by the CHW on THR consumption for herself or for her child, and 0 otherwise.
consumed_THR	Reported consuming or feeding child at least some of the THR received	Binary	Coded 1 if the mother reported that she/her child consumed at least some THR, and 0 otherwise.
**Secondary Outcomes at Beneficiary Level (Infant and Young Child Feeding)**			
advice_exclBF	CHW counselled mother on exclusive breastfeeding of child <6m	Binary	Coded 1 if the mother reported that she was counseled by the CHW on exclusive breastfeeding for children under 6m, and 0 otherwise.
only_bf_0_5m	Child (0-5m) was fed only breastmilk in last 24 hours	Binary	Coded 1 if the mother reported that her child aged 0-5m was only fed breastmilk in the last 24 hours, and 0 otherwise.
advice_CFinitiation	CHW counselled mother on the right time to start complementary feeding	Binary	Coded 1 if the mother reported that she was counseled by the CHW on the right time to start complementary feeding, and 0 otherwise.
iycf_introfood	Child (6-8m) was fed some semi-solid or solid food in last 24 hours	Binary	Coded 1 if the mother reported that her child aged 6-8m was fed any semi-solid or solid food in the last 24 hours, and 0 otherwise.
advice_adequateIYCF	CHW counselled mother on adequate diet (frequency, diet diversity) for child (6-12m)	Binary	Coded 1 if the mother reported that she was counselled by the CHW on adequate diet (number of meals and diet diversity) for her child aged 6-12m, and 0 otherwise.
adeq_diet	Child (6-12m) received adequate diet (number of meals, diet diversity) in last 24 hours	Binary	Coded 1 if the child was fed adequate number of meals with minimum diet diversity for their age as per UN/WHO guidelines, and 0 otherwise.

Name	Description	Type	Notes on Construction and Measurement
**Covariates and Control Variables**			
**Individual and Household Characteristics**			
mo_age	Beneficiary's age in completed years	Continuous	
agemth	Child's age in completed months	Continuous	
child_sex	Sex of child	Binary	0=Male, 1=Female
mo_edu8	Beneficiary's education >= 8 years	Binary	Coded 1 if mother's education level is >= 8 years (elementary school or higher), and 0 otherwise. Missing values recoded as 0 (0.4%)
mo_work	Beneficiary is engaged in work to earn income	Binary	Coded 1 if mother is engaged in job/work to earn income, and 0 otherwise.
SC_ST	Beneficiary belongs to Scheduled Caste/ Scheduled Tribe category	Binary	Coded 1 if mother belongs to SC/ST category, and 0 otherwise. Coded 0 if respondent refused to specify caste (0.09%)
score	PCA Index score for household wealth	Continuous	PCA index score calculated based on ownership of 28 assets, including television, mobile phone, bed/cot, refrigerator, bicycle, AC/cooler, etc.
sleeproom	Number of rooms used for sleeping in the household	Continuous	Number of rooms in the house that are used for sleeping by household members
own_land	Household owns any agricultural land	Binary	Coded 1 if household owns any agricultural land, and 0 otherwise. 'Don't know' recoded as 0 (2%)
im_fuels	Household uses improved fuel for cooking	Binary	Coded 1 if household uses improved fuel for cooking (electricity, LPG, natural gas, or biogas), and 0 otherwise.
hh_toilet	Household has a functional toilet	Binary	Coded 1 if household has a functional toilet, and 0 otherwise.
**CHW and Anganwadi Center (AWC) Characteristics[#]**			
chw_age	CHW's age in completed years	Continuous	
chw_edu12	CHW's education >= 12 years	Binary	Coded 1 if CHW's education level is >= 12 years (high-school or higher), and 0 otherwise. Missing values recoded as 0 (0.3%)
chw_SC_ST	CHW belongs to Scheduled Caste/ Scheduled Tribe category	Binary	Coded 1 if CHW belongs to SC/ST category, and 0 otherwise. Coded 0 if CHW refused to specify caste (0.52%)
chw_experience	Number of years of experience as an CHW	Continuous	
chw_otherwork	CHW is engaged in other work to earn income	Binary	Coded 1 if CHW is engaged in any work/occupation to earn income (other than being CHW), and 0 otherwise.
awc_electricity	AWC has electricity	Binary	Coded 1 if AWC had electricity supply, and 0 otherwise.
blavg_awc_population	Total population covered in the catchment areas of the AWC	Continuous	Total population in AWC's catchment area at the baseline
blavg_awc_water_toilet	AWC had drinking water and toilet on premises at baseline	Binary	Coded 1 if baseline AWC has drinking water and toilet on premises, and 0 otherwise
blavg_awc_scale_chart	AWC had child weighing scale and growth chart at baseline	Binary	Coded 1 if baseline AWC has child weighing scale and growth chart on premises, and 0 otherwise

[#] *In cases where CHW-level data was missing for a few covariates used in the estimation model (1-3% of the AWWs), the missing values were imputed using block-level averages of these covariates. We tested whether such imputation led to biased estimates by including a dummy variable in the estimation models to flag observations which use imputed data and found that this dummy variable was statistically insignificant across all the outcomes.*

Table S2. Pre-Intervention Balance in Covariates using Baseline Data, 2017.

Variables	MP				Bihar			
	N	Comparison	Intervention	Difference	N	Comparison	Intervention	Difference
**Individual and Household Characteristics**								
Beneficiary's age (in completed years)	2229	23.99	24.01	0.02 [-0.3 - 0.33]; p-value 0.922	2764	24.22	24.70	0.48 [0.11 - 0.85]; p-value 0.01
Beneficiary's education (in years)	2228	4.51	4.97	0.43 [0.01 - 0.86]; p-value 0.046	2764	3.89	3.29	-0.6 [-1.08 - -0.13]; p-value 0.013
Beneficiary completed at least 8 years of education	2229	36.60%	39.54%	0.03 [-0.02 - 0.07]; p-value 0.243	2764	29.50%	24.45%	-0.05 [-0.09 - -0.01]; p-value 0.024
Beneficiary works to earn income	2229	44.71%	43.38%	-0.01 [-0.05 - 0.03]; p-value 0.553	2764	10.07%	11.14%	0.01 [-0.02 - 0.04]; p-value 0.454
Beneficiary was married before 18 years of age	1232	26.71%	32.52%	0.06 [0.01 - 0.11]; p-value 0.028	1277	44.53%	46.00%	0.01 [-0.04 - 0.07]; p-value 0.612
Beneficiary owns an Aadhar card	2229	93.92%	94.06%	0 [-0.02 - 0.02]; p-value 0.901	2764	96.98%	95.78%	-0.01 [-0.03 - 0]; p-value 0.09
Beneficiary owns a bank account	2229	78.31%	80.27%	0.02 [-0.02 - 0.06]; p-value 0.318	2764	76.12%	79.55%	0.03 [0 - 0.07]; p-value 0.068
Beneficiary ever used any family planning methods	1235	42.93%	43.55%	0.01 [-0.05 - 0.07]; p-value 0.834	1278	36.56%	34.17%	-0.02 [-0.09 - 0.05]; p-value 0.55
Total number of pregnancies in beneficiary's lifetime	2229	2.42	2.41	-0.01 [-0.13 - 0.11]; p-value 0.871	2764	2.67	2.83	0.16 [0.03 - 0.28]; p-value 0.013
Mother had a normal delivery	1235	92.03%	92.26%	0 [-0.03 - 0.03]; p-value 0.869	1278	91.09%	93.26%	0.02 [-0.01 - 0.05]; p-value 0.16
Beneficiary belongs to scheduled caste/tribe household	2229	55.73%	49.86%	-0.06 [-0.12 - 0]; p-value 0.041	2764	34.89%	38.43%	0.04 [-0.03 - 0.1]; p-value 0.253
Beneficiary belongs to Below-Poverty-Line household	2229	63.40%	65.48%	0.02 [-0.02 - 0.07]; p-value 0.361	2764	57.91%	61.94%	0.04 [-0.01 - 0.09]; p-value 0.094
Beneficiary lives in a pucca house	2229	25.75%	28.86%	0.03 [-0.02 - 0.08]; p-value 0.195	2764	37.70%	37.55%	0 [-0.05 - 0.05]; p-value 0.977
Number of rooms used for sleeping in beneficiary's household	2229	1.81	1.86	0.05 [-0.04 - 0.14]; p-value 0.251	2764	1.87	1.81	-0.06 [-0.13 - 0.02]; p-value 0.162

Variables	MP				Bihar			
	N	Comparison	Intervention	Difference	N	Comparison	Intervention	Difference
Beneficiary's household owns any agricultural land	2229	76.37%	73.70%	-0.03 [-0.07 - 0.02]; p-value 0.259	2764	46.83%	42.94%	-0.04 [-0.09 - 0.01]; p-value 0.14
Beneficiary's household uses improved fuel for cooking	2229	10.05%	13.70%	0.04 [0.01 - 0.07]; p-value 0.012	2764	17.48%	14.85%	-0.03 [-0.06 - 0.01]; p-value 0.14
Beneficiary's household gets drinking water from improved source	2229	13.14%	13.97%	0.01 [-0.03 - 0.04]; p-value 0.623	2764	60.07%	55.75%	-0.04 [-0.1 - 0.01]; p-value 0.119
Beneficiary's household has a functional toilet	2229	51.85%	44.75%	-0.07 [-0.13 - -0.01]; p-value 0.028	2764	23.38%	39.23%	0.16 [0.1 - 0.21]; p-value 0
Household Wealth Index based on Principle Component Analysis	1235	0.48	0.44	-0.05 [-0.34 - 0.24]; p-value 0.74	1278	-0.31	-0.58	-0.27 [-0.52 - -0.02]; p-value 0.037
**Health services to mothers**								
Beneficiary registered pregnancy with the Anganwadi Centres (AWC)	2229	72.13%	66.21%	-0.06 [-0.12 - 0]; p-value 0.056	2764	78.42%	80.93%	0.02 [-0.02 - 0.07]; p-value 0.296
Mother received at least 4 Antenatal check-ups from a health professional/worker	1235	51.38%	49.35%	-0.02 [-0.08 - 0.04]; p-value 0.512	1278	25.63%	19.91%	-0.06 [-0.1 - -0.01]; p-value 0.02
Mother had an institutional delivery	1235	81.63%	81.77%	0 [-0.04 - 0.04]; p-value 0.963	1278	72.50%	66.93%	-0.06 [-0.11 - 0]; p-value 0.062
**Community Health Worker (CHW) Characteristics**								
CHW's age	417	39.33	40.65	1.31 [-0.44 - 3.06]; p-value 0.142	428	39.08	37.52	-1.56 [-3.03 - -0.1]; p-value 0.04
CHW's education (in years)	417	10.11	10.85	0.74 [0.08 - 1.39]; p-value 0.027	428	11.51	11.82	0.3 [-0.09 - 0.69]; p-value 0.13
CHW completed at least 12 years of education	417	41.35%	51.67%	0.1 [0.01 - 0.2]; p-value 0.036	428	53.52%	57.67%	0.04 [-0.06 - 0.14]; p-value 0.42
CHW belongs to Scheduled cate/tribe household	417	52.88%	42.11%	-0.11 [-0.19 - -0.03]; p-value 0.01	428	16.43%	21.40%	0.05 [-0.03 - 0.13]; p-value 0.19
CHW owns a smartphone	417	22.60%	94.26%	0.72 [0.65 - 0.79]; p-value 0	428	3.29%	93.02%	0.9 [0.86 - 0.94]; p-value 0
Number of years of experience as an CHW	417	14.65	15.35	0.71 [-0.79 - 2.21]; p-value 0.352	428	13.89	11.27	-2.62 [-3.89 - -1.35]; p-value 0

Variables	MP				Bihar			
	N	Comparison	Intervention	Difference	N	Comparison	Intervention	Difference
CHW is engaged in other work to earn income	417	3.37%	11.96%	0.09 [0.04 - 0.14]; p-value 0.001	428	1.41%	5.58%	0.04 [0.01 - 0.07]; p-value 0.01
Number of months in the last year for which the CHW did not receive salary	417	0.75	2.03	1.28 [0.68 - 1.88]; p-value 0	428	7.25	6.94	-0.32 [-0.99 - 0.35]; p-value 0.34
CHW is trained in 7 key topics under the systems strengthening programme	417	69.23%	53.11%	-0.16 [-0.25 - -0.07]; p-value 0.001	428	38.97%	39.53%	0 [-0.08 - 0.09]; p-value 0.96
AWC has pucca construction	417	80.29%	84.21%	0.04 [-0.04 - 0.11]; p-value 0.302	428	54.93%	52.56%	-0.02 [-0.11 - 0.07]; p-value 0.62
AWC has drinking water on premises	417	67.79%	57.89%	-0.1 [-0.19 - -0.01]; p-value 0.037	428	44.13%	46.51%	0.02 [-0.07 - 0.12]; p-value 0.62
AWC has toilet on premises	417	38.94%	28.71%	-0.1 [-0.19 - -0.02]; p-value 0.021	428	11.27%	18.14%	0.07 [0 - 0.14]; p-value 0.04
AWC has drinking water and toilet on premises	417	27.88%	18.66%	-0.09 [-0.17 - -0.01]; p-value 0.022	428	7.98%	14.88%	0.07 [0.01 - 0.13]; p-value 0.02
AWC has electricity supply	417	21.15%	15.79%	-0.05 [-0.13 - 0.02]; p-value 0.151	428	16.43%	16.28%	0 [-0.07 - 0.07]; p-value 0.97
AWC has functional child weighing scale	417	92.31%	83.73%	-0.09 [-0.15 - -0.02]; p-value 0.01	428	44.60%	63.26%	0.18 [0.09 - 0.28]; p-value 0
AWC has growth charts for children	417	82.69%	74.64%	-0.08 [-0.16 - -0.01]; p-value 0.036	428	41.78%	49.30%	0.07 [-0.02 - 0.17]; p-value 0.13
AWC has child weighing scale and growth charts for children	417	80.77%	68.90%	-0.12 [-0.2 - -0.04]; p-value 0.004	428	29.58%	43.26%	0.13 [0.05 - 0.22]; p-value 0
CHW reported facing problems with Take-Home-Ration supply from district administration	417	11.06%	14.35%	0.03 [-0.03 - 0.09]; p-value 0.3	428	48.36%	41.40%	-0.07 [-0.16 - 0.02]; p-value 0.11
Total population in AWC catchment area	411	728.17	856.40	124.61 [65.6 - 183.6]; p-value 0	427	1109.19	1116.86	7.86 [-48.97 - 64.68]; p-value 0.79
Total population covered by AWC >= 800	411	38.16%	50.00%	0.11 [0.02 - 0.21]; p-value 0.019	427	91.04%	94.42%	0.03 [-0.02 - 0.08]; p-value 0.19
Number of pregnant women in AWC catchment area	411	7.35	7.69	0.32 [-0.84 - 1.47]; p-value 0.59	424	12.30	12.39	0.1 [-0.8 - 0.99]; p-value 0.83

Variables	MP				Bihar			
	N	Comparison	Intervention	Difference	N	Comparison	Intervention	Difference
Number of lactating women in AWC catchment area	411	5.87	7.37	1.5 [0.68 - 2.32]; p-value 0	424	12.08	11.07	-1 [-1.98 - -0.02]; p-value 0.04
**Outcomes**								
% of mothers who received adequate number of home visits in last 3 months	1235	17.07%	14.52%	-0.03 [-0.07 - 0.02]; p-value 0.249	1278	5.94%	8.62%	0.03 [0 - 0.06]; p-value 0.09
% of mothers who received at least 50% of the correct counselling messages as per their life stage	1235	8.13%	7.74%	0 [-0.04 - 0.03]; p-value 0.815	1278	1.25%	2.19%	0.01 [-0.01 - 0.02]; p-value 0.228
% of pregnant women who received adequate number of home visits in last 3 months	994	38.73%	41.05%	0.02 [-0.05 - 0.1]; p-value 0.544	1486	23.47%	29.62%	0.06 [0 - 0.11]; p-value 0.036
% of pregnant women who received at least 50% of the correct counselling messages as per their life stage	994	0.96%	1.68%	0.01 [-0.01 - 0.02]; p-value 0.421	1486	1.87%	1.77%	0 [-0.02 - 0.02]; p-value 0.898
% of mothers who reported that their child was weighed by CHW at least once in last 3 months	1235	50.57%	57.10%	0.07 [0 - 0.13]; p-value 0.05	1278	15.00%	23.98%	0.09 [0.03 - 0.14]; p-value 0.002
% of mothers who reported that CHW discussed their child's weight or showed child's growth chart	1235	34.96%	37.90%	0.03 [-0.03 - 0.09]; p-value 0.328	1278	9.84%	16.77%	0.07 [0.02 - 0.11]; p-value 0.004
% of mothers who received THR at least once a month from CHW	1235	73.33%	74.35%	0.01 [-0.04 - 0.06]; p-value 0.701	1278	22.81%	17.71%	-0.05 [-0.1 - 0]; p-value 0.05
% of mothers (6-12m child) who recall being counselled about mother's/child's THR consumption by CHW in last 3 months	470	11.06%	6.15%	-0.05 [-0.11 - 0]; p-value 0.069	454	0.43%	2.70%	0.02 [0 - 0.05]; p-value 0.061
% of mothers who reported that they/their child consumed any THR	1235	78.21%	80.16%	0.02 [-0.03 - 0.07]; p-value 0.411	1278	59.38%	42.79%	-0.17 [-0.23 - -0.1]; p-value 0
% of mothers (0-6m child) who recalled being counselled about exclusive breastfeeding by CHW	765	17.74%	18.88%	0.01 [-0.05 - 0.07]; p-value 0.672	824	5.64%	9.86%	0.04 [0 - 0.08]; p-value 0.041
% of children aged 0-6m who were only fed breastmilk during the previous 24 hours	765	46.02%	49.47%	0.03 [-0.04 - 0.1]; p-value 0.364	824	60.05%	51.44%	-0.09 [-0.15 - -0.02]; p-value 0.012
% of mothers (6-8m child) who recalled being counselled about the right time to start complementary feeding by CHW	265	15.08%	17.99%	0.02 [-0.07 - 0.11]; p-value 0.698	272	1.60%	9.52%	0.08 [0.03 - 0.14]; p-value 0.004
% of children aged 6-8m who received solid, semi-solid, or soft foods during previous 24 hours	265	68.25%	57.55%	-0.12 [-0.23 - -0.01]; p-value 0.037	272	71.20%	63.27%	-0.08 [-0.19 - 0.03]; p-value 0.133

Variables	MP				Bihar			
	N	Comparison	Intervention	Difference	N	Comparison	Intervention	Difference
% of mothers (6-12m child) who recalled being counselled about dietary diversity and adequate frequency of meals for their child by CHW	470	17.70%	17.21%	-0.01 [-0.08 - 0.06]; p-value 0.828	454	3.45%	7.66%	0.04 [0 - 0.08]; p-value 0.064
% of children aged 6-12m who received adequate diet as per their age during previous 24 hours	470	7.96%	10.66%	0.03 [-0.03 - 0.08]; p-value 0.331	454	4.31%	6.76%	0.02 [-0.02 - 0.07]; p-value 0.269

Table S3. Respondent, Household and CHW Characteristics at Endline, 2019.

Variables	MP				Bihar			
	N	Comparison	Intervention	Difference	N	Comparison	Intervention	Difference
**Individual and Household Characteristics**								
Beneficiary's age (in completed years)	4266	24.03	23.88	-0.15 [-0.4 - 0.11]; p-value 0.249	4767	24.28	24.69	0.42 [0.14 - 0.69]; p-value 0.003
Beneficiary's education (in years)	4266	5.32	5.49	0.11 [-0.21 - 0.43]; p-value 0.5	4731	4.48	4.03	-0.45 [-0.84 - -0.05]; p-value 0.027
Beneficiary completed at least 8 years of education	4266	44.24%	44.54%	0 [-0.04 - 0.03]; p-value 0.873	4767	35.15%	29.92%	-0.05 [-0.09 - -0.02]; p-value 0.005
Beneficiary works to earn income	4266	25.73%	25.35%	0 [-0.04 - 0.04]; p-value 0.982	4767	8.81%	11.12%	0.03 [0.01 - 0.05]; p-value 0.013
Beneficiary was married before 18 years of age	4266	21.13%	29.84%	0.09 [0.06 - 0.12]; p-value 0	4767	46.39%	49.52%	0.03 [0 - 0.06]; p-value 0.048
Mother spent substantial portion of pregnancy in her maternal home	3218	27.64%	33.47%	0.06 [0.02 - 0.1]; p-value 0.007	3417	41.86%	40.49%	-0.01 [-0.05 - 0.03]; p-value 0.553
Beneficiary finds it easy to reach the Anganwadi Center (AWC)	4266	83.08%	81.65%	-0.01 [-0.05 - 0.02]; p-value 0.395	4767	76.43%	79.06%	0.03 [-0.01 - 0.06]; p-value 0.131
Beneficiary owns an Aadhar card	4266	97.82%	97.28%	-0.01 [-0.02 - 0]; p-value 0.244	4767	96.60%	96.39%	0 [-0.01 - 0.01]; p-value 0.67
Beneficiary owns a bank account	4266	90.85%	90.07%	-0.01 [-0.03 - 0.01]; p-value 0.334	4767	84.14%	86.99%	0.03 [0 - 0.05]; p-value 0.03
Beneficiary ever used any family planning methods	4266	34.93%	31.76%	-0.03 [-0.07 - 0]; p-value 0.051	4767	14.60%	19.35%	0.05 [0.03 - 0.08]; p-value 0
Total number of pregnancies in beneficiary's lifetime	4266	2.39	2.32	-0.06 [-0.16 - 0.03]; p-value 0.164	4767	2.70	2.92	0.21 [0.11 - 0.32]; p-value 0
Mother had a normal delivery	3218	91.59%	91.87%	0 [-0.02 - 0.02]; p-value 0.691	3417	88.50%	91.73%	0.03 [0.01 - 0.05]; p-value 0.004
Child was born on time	3218	95.60%	94.62%	-0.01 [-0.03 - 0.01]; p-value 0.35	3417	92.47%	88.50%	-0.04 [-0.06 - -0.01]; p-value 0.002
Beneficiary belongs to Scheduled caste/tribe household	4266	55.91%	51.09%	-0.04 [-0.09 - 0]; p-value 0.055	4767	35.95%	36.59%	0.01 [-0.04 - 0.06]; p-value 0.752

Variables	MP				Bihar			
	N	Comparison	Intervention	Difference	N	Comparison	Intervention	Difference
Beneficiary belongs to Below-Poverty-Line household	4266	70.11%	68.46%	-0.01 [-0.05 - 0.02]; p-value 0.384	4767	60.11%	59.13%	-0.01 [-0.05 - 0.02]; p-value 0.416
Beneficiary lives in a pucca house	4266	33.20%	38.04%	0.05 [0.01 - 0.08]; p-value 0.012	4767	52.68%	46.45%	-0.05 [-0.09 - -0.02]; p-value 0.006
Number of rooms used for sleeping in beneficiary's household	4266	2.08	2.11	0.03 [-0.06 - 0.11]; p-value 0.511	4767	2.21	2.12	-0.08 [-0.17 - 0.01]; p-value 0.073
Beneficiary's household owns any agricultural land	4266	74.02%	70.69%	-0.03 [-0.07 - 0.01]; p-value 0.178	4767	42.83%	40.54%	-0.02 [-0.06 - 0.02]; p-value 0.376
Beneficiary's household uses improved fuel for cooking	4266	15.34%	16.17%	0.01 [-0.02 - 0.04]; p-value 0.569	4767	23.41%	24.09%	0.01 [-0.03 - 0.04]; p-value 0.691
Beneficiary's household gets drinking water from improved source	4266	24.99%	26.10%	0.01 [-0.02 - 0.04]; p-value 0.525	4767	70.30%	66.39%	-0.05 [-0.08 - -0.01]; p-value 0.016
Beneficiary's household has a functional toilet	4266	73.82%	61.87%	-0.12 [-0.16 - -0.07]; p-value 0	4767	36.91%	47.88%	0.11 [0.06 - 0.15]; p-value 0
Household Wealth Index based on Principle Component Analysis	4266	0.45	0.37	-0.08 [-0.29 - 0.12]; p-value 0.426	4767	-0.30	-0.43	-0.14 [-0.28 - 0.01]; p-value 0.063
**Health services to mothers**								
Beneficiary registered pregnancy with the AWC	4266	81.59%	80.09%	-0.01 [-0.04 - 0.02]; p-value 0.409	4767	72.48%	74.11%	0.01 [-0.02 - 0.05]; p-value 0.377
Beneficiary attended a Village Health and Nutrition Day in last three months (or since birth)	4266	49.98%	56.44%	0.06 [0.02 - 0.1]; p-value 0.004	4767	22.44%	25.18%	0.03 [-0.01 - 0.06]; p-value 0.125
Mother received at least 4 Antenatal check-ups from a health professional/worker	3218	61.04%	57.56%	-0.04 [-0.08 - 0]; p-value 0.076	3417	23.64%	15.32%	-0.08 [-0.11 - -0.05]; p-value 0
Mother had institutional delivery	3218	85.50%	85.95%	0 [-0.03 - 0.04]; p-value 0.919	3417	78.05%	69.48%	-0.08 [-0.13 - -0.04]; p-value 0
Number of visits to mother in last three months by volunteer (called ASHA) of another government programme under rural health mission	3218	1.74	1.97	0.22 [0.05 - 0.38]; p-value 0.01	3417	1.29	1.24	-0.05 [-0.2 - 0.1]; p-value 0.477
**Community Health Worker (CHW) Characteristics**								
CHW's age	684	38.06	39.66	1.5 [0.13 - 2.86]; p-value 0.032	633	39.33	37.67	-1.64 [-2.92 - -0.37]; p-value 0.012

Variables	MP				Bihar			
	N	Comparison	Intervention	Difference	N	Comparison	Intervention	Difference
CHW's education (in years)	684	10.64	10.93	0.14 [-0.41 - 0.69]; p-value 0.623	633	11.50	11.80	0.3 [-0.03 - 0.64]; p-value 0.078
CHW completed at least 12 years of education	684	48.44%	54.68%	0.05 [-0.03 - 0.13]; p-value 0.215	633	55.10%	57.52%	0.02 [-0.06 - 0.1]; p-value 0.572
CHW belongs to scheduled caste/tribe household	684	58.64%	47.73%	-0.05 [-0.11 - 0.01]; p-value 0.091	633	14.97%	20.06%	0.05 [0 - 0.11]; p-value 0.073
CHW owns a smartphone	684	32.58%	100.00%	0.67 [0.62 - 0.72]; p-value 0	633	59.18%	94.69%	0.35 [0.28 - 0.42]; p-value 0
Number of years of experience as an CHW	684	14.31	15.73	1.42 [0.18 - 2.66]; p-value 0.025	633	15.59	12.17	-3.37 [-4.51 - -2.23]; p-value 0
CHW is engaged in other work to earn income	684	5.38%	6.04%	0 [-0.04 - 0.04]; p-value 0.916	633	7.48%	8.55%	0.01 [-0.04 - 0.05]; p-value 0.759
Number of months in the last year for which the CHW did not receive salary	684	1.42	0.98	-0.45 [-0.79 - -0.1]; p-value 0.012	633	5.30	4.53	-0.81 [-1.24 - -0.39]; p-value 0
CHW is trained in 7 key topics under the systems strengthening programme	684	75.35%	74.62%	-0.04 [-0.1 - 0.02]; p-value 0.218	633	53.06%	51.92%	-0.01 [-0.09 - 0.07]; p-value 0.851
AWC has pucca construction	684	80.45%	90.63%	0.09 [0.04 - 0.15]; p-value 0.001	633	58.16%	49.85%	-0.07 [-0.14 - 0]; p-value 0.041
AWC has drinking water on premises	681	65.91%	67.78%	0.01 [-0.06 - 0.08]; p-value 0.769	611	49.31%	49.54%	0 [-0.09 - 0.08]; p-value 0.917
AWC has toilet on premises	681	52.84%	48.63%	-0.06 [-0.14 - 0.01]; p-value 0.096	611	18.40%	24.15%	0.05 [-0.01 - 0.12]; p-value 0.107
AWC has drinking water and toilet on premises	684	40.23%	38.67%	-0.03 [-0.11 - 0.04]; p-value 0.408	633	14.97%	17.70%	0.02 [-0.04 - 0.08]; p-value 0.46
AWC has electricity supply	684	32.58%	19.05%	-0.14 [-0.2 - -0.07]; p-value 0	633	25.51%	27.26%	0.01 [-0.06 - 0.09]; p-value 0.72
AWC has functional child weighing scale	684	84.42%	83.38%	-0.02 [-0.09 - 0.04]; p-value 0.459	633	75.85%	83.48%	0.07 [0.01 - 0.14]; p-value 0.033
AWC has growth charts for children	684	45.61%	50.15%	0.02 [-0.06 - 0.1]; p-value 0.668	633	38.44%	35.40%	-0.03 [-0.11 - 0.05]; p-value 0.464

Variables	MP				Bihar			
	N	Comparison	Intervention	Difference	N	Comparison	Intervention	Difference
AWC has child weighing scale and growth charts for children	684	43.06%	47.73%	0.02 [-0.06 - 0.1]; p-value 0.623	633	34.01%	33.92%	0 [-0.08 - 0.08]; p-value 0.975
CHW reported facing problems with Take-Home-Ration supply from district administration	684	12.46%	7.25%	-0.06 [-0.11 - -0.01]; p-value 0.012	633	35.71%	53.39%	0.17 [0.09 - 0.26]; p-value 0
Total population in AWC catchment area	613	698.11	829.11	117.92 [65.67 - 170.17]; p-value 0	534	1081.78	1023.97	-57.95 [-102.5 - -13.4]; p-value 0.011
Total population covered by AWC >= 800	613	37.74%	51.16%	0.11 [0.03 - 0.2]; p-value 0.009	534	91.44%	86.86%	-0.05 [-0.1 - 0.01]; p-value 0.097
Number of pregnant women in AWC catchment area	612	5.83	7.72	1.91 [1.21 - 2.62]; p-value 0	544	12.15	11.48	-0.68 [-1.36 - 0]; p-value 0.051
Number of lactating women in AWC catchment area	612	6.92	8.60	1.67 [0.89 - 2.46]; p-value 0	543	12.57	9.88	-2.67 [-3.39 - -1.95]; p-value 0
CHW has frequent interactions with her immediate supervisor	684	49.86%	53.47%	0.05 [-0.03 - 0.13]; p-value 0.233	633	60.54%	54.57%	-0.06 [-0.15 - 0.02]; p-value 0.156
